# The regulation of cyclin D1 degradation: roles in cancer development and the potential for therapeutic invention

**DOI:** 10.1186/1476-4598-6-24

**Published:** 2007-04-02

**Authors:** John P Alao

**Affiliations:** 1 Department of Cell and Molecular Biology, Lundberg Laboratory, Gothenburg University, P. O. Box 462, S-405 30, Gothenburg, Sweden

## Abstract

Cyclin D1 is an important regulator of cell cycle progression and can function as a transcriptionl co-regulator. The overexpression of cyclin D1 has been linked to the development and progression of cancer. Deregulated cyclin D1 degradation appears to be responsible for the increased levels of cyclin D1 in several cancers. Recent findings have identified novel mechanisms involved in the regulation of cyclin D1 stability. A number of therapeutic agents have been shown to induce cyclin D1 degradation. The therapeutic ablation of cyclin D1 may be useful for the prevention and treatment of cancer. In this review, current knowledge on the regulation of cyclin D1 degradation is discussed. Novel insights into cyclin D1 degradation are also discussed in the context of ablative therapy. A number of unresolved questions regarding the regulation of cellular cyclin D1 levels are also addressed.

## Background

The cyclin D1 proto-oncogene is an important regulator of G1 to S phase progression in many different cell types. Together with its binding partners cyclin dependent kinase 4 and 6 (CDK4 and CDK6), cyclin D1 forms active complexes that promote cell cycle progression by phosphorylating and inactivating the retinoblastoma protein (RB) [1-3]. More recent studies have demonstrated that cyclin D1 also functions as transcriptional modulator by regulating the activity of several transcription factors and histone deacetylase (HDAC3) (reviewed in [4]). This activity is independent of CDK4 activity. The cyclin D1 protein has been shown to be unstable with a short half-life (~24 min) [5,6] and is degraded mainly via the 26S proteasome in a ubiquitin-dependent manner [6]. Early studies suggested that the Skp2 F-box protein might be involved in cyclin D1 degradation [7]. Recently, two further F-box proteins were identified in separate studies as playing major roles in targeting the cyclin for degradation [8,9].

Cyclin D1 is important for the development and progression of several cancers including those of the breast, oesophagus, bladder and lung [10-19]. Overexpression of cyclin D1 has also been linked to the development of endocrine resistance in breast cancer cells [20-22]. Cyclin D1 overexpression is a common event in cancer but does not occur solely as a consequence of gene amplification. Rather, increased levels of cyclin D1 frequently result from its defective regulation at the post-translational level [23,24]. A number of therapeutic agents have been observed to induce cyclin D1 degradation *in vitro *[25-30]. These studies indicate that the induction of cyclin D1 degradation may offer a useful avenue for therapeutic intervention [25-32].

In this review, current knowledge on the regulation of cyclin D1 degradation is discussed with a particular emphasis on recent discoveries in this area. The roles of cyclin D1 as a regulator of cell cycle progression have been extensively reviewed [1,4,15,33-37] and will only be mentioned in the context of its degradation. Here, the current knowledge on the regulation of cell cycle-dependent and drug induced cyclin D1 degradation is reviewed. The discovery of novel regulators of cyclin D1 stability [8,9,27] and their impact on this area of research is also examined.

### Cell cycle phase-dependent degradation of cyclin D1

**Figure 1:**
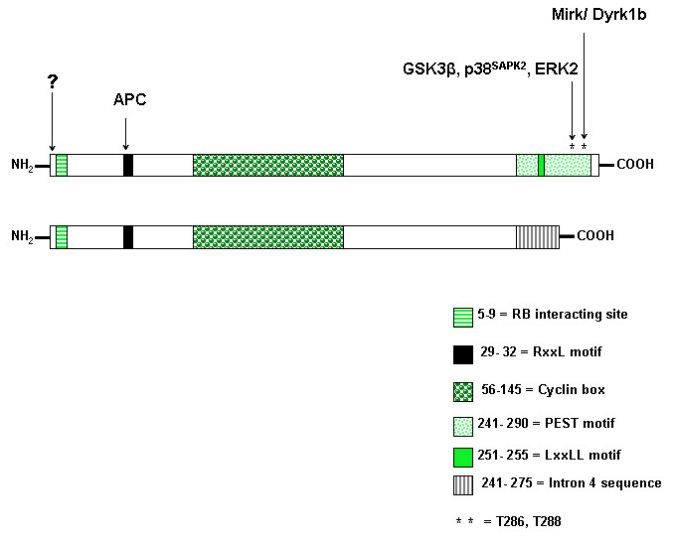
Schematic representation of cyclin D1 (top) and cyclin D1b (bottom) regulatory sequences. Cyclin D1 stability is regulated by various mechanisms. The n-terminal region has recently been shown to be important for regulating stability [27]. (**?**) The mechanisms that regulate cyclin D1 stability via the n-terminal remain to be clearly defined. The RxxL motif is required for APC (Anaphase Promoting Complex) mediated degradation following genotoxic insult [48]. GSK3β phosphorylates threonine residue 286 (T286) and regulates cyclin D1 nuclear export and stability [5, 40]. p38^SAPK2 ^and ERK2 have also been shown to regulate cyclin D1 stability by phosphorylating T286 [9, 49, 114]. The threonine 288 residue (T288) has also been shown to regulate cyclin D1 stability. Phosphorylation of T288 is mediated by the mirk/Dyrk 1b kinase [47]. In cyclin D1b, regulatory motifs and residues within the c-terminal region downstream of residue 240 are replaced by sequence from intron 4 of the *CCND1 *gene [62, 63]. Adapted from Knudsen, 2006 [13, 65].

**Figure 2:**
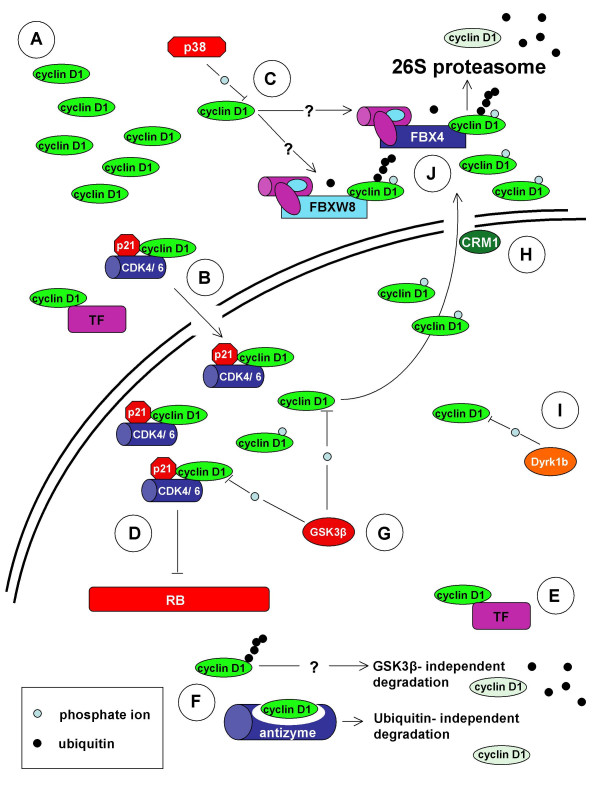
Regulation of cyclin D1 degradation. **A. **Cyclin D1 does not contain a nuclear localization signal (NLS) [159] and its sequestration may result in accumulation within the cytoplasm [45, 75, 76]. **B. **Cytoplasmic cyclin D1 is transported into the nucleus in association with its binding partners e.g CDK4 and possibly various transcription factors (TF) [159-161]. **C. **p38^SAPK2 ^has been shown to phosphorylate cyclin D1 on threonine residue 286 (T286) and induce its proteasomal degradation [49, 114]. It is unclear if the F-box proteins FBX4 and FBXW8 are involved in mediating p38^SAPK2 ^induced cyclin D1 degradation in the cytoplasm. **D, E. **Within the nucleus, active cyclin dependent kinase 4 (CDK4) or CDK6- cyclin D1 complexes phosphorylate the retinoblastoma protein (RB) [2]. Cyclin D1 can also influence the activity of various transcription factors independently of CDK4/6 [4]. **F. **Free cyclin D1 is degraded through the ubiquitin dependent 26S proteasomal degradation pathway independently of glycogen synthase kinase 3β (GSK3β) [46]. Antizyme can also mediate cyclin D1 degradation via the 26S proteasome independently of ubiquitin [56]. **G, H. **GSK3β phosphorylates cyclin D1 on T286 which facilitates its nuclear export by the exportin CRM1. GSK3β influences cyclin D1 stability since the phosphorylated form of the cyclin is subsequently degraded within the cytoplasm [5, 6, 40]. **I. **Phosphorylation of T288 is mediated by the **mirk/Dyrk 1b **kinase and can induce cyclin D1 degradation [47]. **J. **FBX4 and FBXW8 ubiquitylate phosphorylated cyclin D1 within the cytoplasm, targeting it for 26S proteasomal degradation [8, 9].

Cyclin D1 levels begin to rise early in G1 and continue to accumulate until the G1/S-phase boundary when levels rapidly decline. The degradation of the cyclin is essential for the replication of DNA because acute overexpression of cyclin D1 in fibroblasts prevented S-phase entry [38,39]. Cyclin D1 has been shown to repress DNA replication by binding to proliferating cell nuclear antigen (PCNA) and Cdk2. The binding of cyclin D1 to PCNA directly inhibits DNA synthesis [38]. Initial studies by Diehl *et al*., [6] demonstrated that cyclin D1 turnover was dependent on threonine 286 (T286) phosphorylation and regulated by ubiquitin-dependent proteasomal degradation. Phosphorylation of cyclin D1 was enhanced by binding to CDK4 and mutation of T286 to alanine (T286A) resulted in greatly increased stability of the cyclin. These studies also showed that CDK4 is not required for T286 phosphorylation, indicating the involvement of an additional kinase. Glycogen synthase kinase 3β (GSK3β) was eventually identified as being capable of phosphorylating cyclin D1 on T286 and inducing its rapid turnover [5]. GSK3β was also shown to promote the redistribution of cyclin D1 from the nucleus to the cytoplasm. It had been noted previously, that cyclin D1 degradation at S- phase was accompanied by its relocation to the cytoplasm [33] while GSK3β levels were observed to increase in the nucleus specifically during S-phase [6]. In addition, the highly stable T286A mutant maintained a nuclear localization pattern throughout the cell cycle. Further work eebsequently demonstrated that phosphorylation of T286 facilitated cyclin D1 nuclear export by enhancing its association with CRM1, a nuclear exportin [40]. Together these findings suggested that GSK3β-dependent phosphorylation of cyclin D1 mediated its nuclear export and rapid degradation within the cytoplasm. Since GSK3β is negatively regulated by the Ras- phosphatidylinositol 3 kinase – Akt pathway [41,42], these findings also linked cyclin D1 stability to mitogenic stimulation (see Figure 1 and 2). Based on their observations, Diehl *et al*., [5] predicted that the deregulation of phosphorylation dependent cyclin D1 degradation might contribute to the development of cancer. Further studies demonstrated that the constitutive overexpression of the T286A mutant but not wild type cyclin D1, resulted in formation of foci in late passage cells. Overexpression of cyclin D1 T286A also facilitated anchorage independent growth of NIH-3T3 cells in soft agar [40].

A number of recent findings have questioned the role of GSK3β in mediating cyclin D1 degradation. Guo *et al*., confirmed the role of T286 phosphorylation in mediating cyclin D1 degradation at S phase and demonstrated that the levels of phosphorylated protein are elevated during this phase of the cell cycle [43,44]. The authors reported however, that there was no change in the activity of GSK3β, Akt or PI3K during S-phase and inhibition of GSK3β activity did not influence cyclin D1 phosphorylation or protein levels during the cell cycle. Similarly, GSK3β localization was not observed to vary with cell cycle expression in MCF-7 breast cancer cells and inhibition of GSK3β activity did not completely abolish cyclin D1 degradation [26,45]. The experimental evidence however, clearly shows that GSK3β can phosphorylate cyclin D1 and inhibition of this activity diminishes the efficiency of cyclin D1 degradation. The impact of differential experimental systems on the conflicting observations described in these studies remains uncertain. Further studies on the role of GSK3β-dependent and independent (see below) cyclin D1 degradation will thus be required.

### GSK3β-independent cyclin D1 degradation

Cyclin D1 degradation has also been shown to occur independently of GSK3β. Studies by Germain *et al*., [46] demonstrated that the cyclin D1 T286A mutant is subject to ubiquitin-dependent degradation. Similarly, a mutant unable to bind CDK4 (cyclin D1-KE) was also ubiquitylated. Since cyclin D1 mutants lacking T286 and/or T288 were still susceptible to ubiquitylation and degradation, the authors proposed the existence of a second pathway that does not require phosphorylation on T286. Since cyclin D1 mutants that fail to bind CDK4 were also subject to ubiquitin-dependent degradation, it appears that this pathway may serve to regulate the cellular levels of free cyclin D1.

More recently, a novel arginine-directed protein kinase has been implicated in the regulation of cyclin D1 stability [47]. Mirk/Dyrk1b is active at G0 and early G1 phases of the cell cycle and phosphorylates cyclin D1 on T288. Knockdown of Mirk/Dyrk1b by siRNA oligos resulted in increased levels of cyclin D1 protein but did not affect mRNA levels. Furthermore, Mirk/Dyrk1b mediated cyclin D1 downregulation was not sensitive to inhibition by lithium chloride (LiCl), an inhibitor of GSK3β. These findings suggest that Mirk/Dyrk1b may cooperate with GSK3β to regulate cyclin D1 levels [47]. Since cyclin D1 degradation occurs mainly at the G1/S phase boundary however, the impact of a kinase active only at G0/G1 is likely to be minimal in proliferating cells. On the other hand, Mirk/Dyrk1b may serve to regulate cyclin D1 levels in situations where GSK3β activity is absent.

In mammalian cells, DNA damage, environmental stress and viral infection have also been shown to induce the ubiquitin-dependent degradation of cyclin D1. Genotoxic stress induces a G1 cell cycle arrest that is mediated by cyclin D1 degradation [48]. Following exposure to ionizing radiation (IR) cyclin D1 is rapidly degraded via the ubiquitin pathway. This degradation differs from the normal cell cycle related proteolysis, since it requires an RxxL destruction box within the N- terminus of cyclin D1. IR induced cyclin D1 degradation requires the Anaphase-Promoting Complex (APC) but occurs independently of GSK3β [48]. Casanovas *et al*., [49] demonstrated that in addition to genotoxic stress, various chemically induced environmental stresses also induce cyclin D1 degradation in mantle cell lymphoma derived Granta 519 cells. Osmotic stress induced by sodium chloride (NaCl), calcium chloride (CaCl_2_) or magnesium chloride (MgCl_2_) resulted in the rapid proteolysis of cyclin D1. Similarly, hydrogen peroxide (H_2_O_2_) induced oxidative stress and sodium arsenite (NaAsO_2_) also induced a rapid fall in cellular cyclin D1 levels. Specific inhibition of 26S proteasomes inhibited cyclin D1 degradation in all cases. Further studies demonstrated that the osmotic stress induced activation of p38^SAPK2 ^results in the phosphorylation of T286 and induces cyclin D1 degradation. Accordingly, specific inhibition of p38^SAPK2 ^with the p38 inhibitor SB203580 abolished osmotic stress but not H_2_O_2 _or NaAsO_2 _mediated cyclin D1 degradation. *In vitro *assays demonstrated that active immunoprecipitated p38^SAPK2 ^from Molt-4 lymphoblastoid cells, phosphorylated cyclin D1 but not CDK2, CDK4 or cyclin A. Similar results were obtained using purified p38^SAPK2 ^and cyclin D1 phosphorylation was abolished by the addition of SB203580 in these assays. p38^SAPK2 ^was also shown to phosphorylate residues T156 and T286A *in vitro*. Inhibition of p38^SAPK2 ^or mutation of cyclin D1 residue T286 to alanine abolished cyclin D1 ubiquitylation. It is thus clear, that osmotic stress induced by compounds such as NaCl results in the ubiquitin-dependent degradation of T286 phosphorylated cyclin D1. This pathway differs from that regulating normal S phase associated degradation, since p38^SAPK2 ^and not GSK3β mediates cyclin D1 phosphorylation. The p38^SAPK2 ^pathway is activated in response to various environmental stresses including UV radiation and histone deacetylase (HDAC) inhibitors (discussed below) [50]. Kidney derived cell lines exposed to high osmolarity transiently undergo cell cycle arrest [51,52]. Following exposure to environmental stress or DNA damage, the rapid degradation cyclin D1 may be necessary to ensure rapid cell cycle arrest. Indeed, the failure to accumulate cyclin D1 during G2 has been shown to prevent entry into a subsequent round of cell division [53,54]. The elevation of cyclin D1 levels in G2 is dependent on external stimuli. The regulation of cyclin D1 accumulation just prior to mitosis, serves to prevent further rounds of cell cycle progression under unfavourable conditions [54]. p38^SAPK2 ^may thus play a central role in mediating cyclin D1 degradation following exposure to environmental stress or genotoxic insult.

Infection of HeLa cells by the Coxsackievirus group B3 (CVB3) has been shown to induce cyclin D1 degradation via the ubiquitin pathway [55]. Inhibition of GSK3β activity by LiCl did not prevent CVB3-induced cyclin D1 degradation. ODC-antizyme, a regulator of ornithine decarboxylase (ODC) has also been shown to target cyclin D1 for degradation via the 26S proteasome [56]. Interestingly, antizyme mediated cyclin D1 degradation occurs independently of ubiquitin or T286 phosphorylation. Upregulation of antizyme resulted in reduced levels of cyclin D1 and has been shown to inhibit the proliferation of transformed NIH3T3 cells and malignant keratinocytes. Similarly, tumour development and growth were also inhibited under conditions of increased antizyme activity [57-59]. It is clear that efficient cyclin D1 degradation can occur independently of GSK3β mediated T286 phosphorylation. The above cited studies also indicate that T286 phosphorylation independent degradation plays an important role in regulating cyclin D1 stability in response to environmental insults (see Figure 1 and 2).

### Cyclin D1b localization, stability and role in cancer development

A cyclin D1 splice variant (cyclin D1b) has been identified in various cell lines and tissues [60-63]. An alternative splicing event within exon 4, results in a 274 amino acid product lacking the C-terminal PEST domain and residue T286. Despite the loss of this region which is known to regulate cyclin D1 stability, the half-life of cyclin D1d is only slightly greater than that of full length variant (cyclin D1a). Similarly to the T286A mutant, cyclin D1b is constitutively nuclear but does accumulate to levels above those of cyclin D1a. Interestingly, cyclin D1b has been shown to be a poor activator of CDK4 and RB phosphorylation [63]. None the less, stable overexpression of cyclin D1b in NIH-3T3 cells results in the formation of foci after 12 passages [63] and promotes anchorage independent growth [62]. Injection of late passage NIH-3T3 cells expressing cyclin D1b into SCID mice resulted in tumour formation [62]. Similar experiments using NIH-3T3 cells expressing cyclin D1a did result in tumour formation. Given the fact that cyclin D1b is relatively unstable and a poor inactivator of RB, its transforming activity is surprising. It is possible that the constitutive nuclear localization, increased mobility or structural changes to the C-terminus facilitate cyclin D1b transforming activity [62-64]. The absence of a c-terminal PEST sequence or T286 residue suggests that the regulation of cyclin D1b is likely to be substantially different from cyclin D1a (see Figure 1 and 2). Both variants may however, be jointly regulated by phosphorylation independent degradation and other pathways [27,46,56]. Cyclin d1b has been reviewed in [13,65].

### Role of the SCF complex in cyclin D1 degradation

The Skp- Cullin- F-box (SCF) complexes regulate protein stability by targeting substrate proteins for ubiquitin-dependent degradation. Cullin 1 (CUL-1) or cullin 7 (CUL-7) provides a scaffold that allows recruitment of the E3 ligase subunit RBX1/ROC1. SKP1 in turn binds the specificity determining component that comprises the F-box protein family. F-box proteins are evolutionally conserved and multiple isoforms from several distinct classes occur in all cell types. The SCF complex has been extensively linked to the ubiquitin-dependent degradation of numerous cell cycle regulators including cyclin D1 [66-70]. Early studies indicated a role for CUL-1 and the SKP2 F-box protein in mediating cyclin D1 ubiquitylation [7]. Inhibition of SKP1, CUL-1 or SKP2 induced the accumulation of cyclin D1 and p21. Furthermore, both cyclin D1 and p21 were shown to interact with CUL-1 in immunoprecipitates from cell lysates. These studies did not however, demonstrate a direct interaction between cyclin D1 and SKP2 or the *in vitro *ubiquitylation of cyclin D1 by SKP2.

More recently, the F-box proteins FBX4 and FBXW8 have been identified as *bona fide *mediators of cyclin D1 ubiquitylation using both *in vitro *and *in vivo *assays [8,9]. FBX4 recognition of cyclin D1 requires T286 phosphorylation and the presence of αB crystallin. Both FBX4 and αB crystallin are required for the rapid ubiquitin-dependent degradation of T286 phosphorylated cyclin D1 and depletion of either component results in the increased stability and accumulation of cyclin D1 [8]. FBX4 and αB crystallin are both cytoplasmic proteins. The nuclear export of T286 phosphorylated cyclin D1 during S phase thus facilitates association with the SCF^FBX4-αB crystallin ^E3 ligase complex. Importantly, a number of human cancer cell lines with high cyclin D1 levels were shown not to express αB crystallin. Exogenously expressed αB crystallin was able to restore the rapid degradation of cyclin D1 in the MCF-7 breast cancer cell line. Low or absent expression of αB crystallin and FBX4 mRNA was also observed in numerous tumour samples from a diverse array of malignancies. Together these findings suggest that deregulated FBX4 activity and/or αB crystallin expression may be responsible for the increase in cyclin D1 stability observed in some cancers [8].

An independent study by Okabe *et al*., [9] has identified FBXW8 as a second cyclin D1 E3 ligase. Interestingly, this study also identified the mitogen activated protein kinase (MAPK) ERK2 as a novel protein kinase capable of phosphorylating cyclin D1 on T286. ERK2 mediated phosphorylation of T286 requires a conserved D-domain (amino acid residues 179–193) within cyclin D1. *In vitro *assays demonstrated that ERK2 phosphorylates wild type but not D-domain deleted (ΔD) or T286A mutant cyclin D1 *in vitro*. Similarly, culture of HCT116 colon cancer cells with the MEK inhibitor U0126 extended the half life of cyclin D1 and abolished T286 phosphorylation. The study did not however, examine the effect of prolonged MEK inhibition by U0126 on cyclin D1 accumulation. In our studies, treatment of MCF-7 cells with U0126 resulted in decreased cyclin D1 protein levels following a 24 h exposure (J. P. Alao *et al*., unpublished observations). ERK2 mediated regulation of cyclin D1 stability may thus be cell line specific. The study by Okabe *et al*., [9] also identified FBXW8 as a second F-box protein capable of mediating cyclin D1 polyubiquitylation. Cyclin D1 associated with Skp1, cullin (CUL1 and CUL7) and CDK4. FBXW8 interacted with cyclin D1 in a T286 phosphorylation-dependent manner and mediated its ubiquitylation *in vitro*. FBXW8 mediated ubiquitylation of cyclin D1 was enhanced by the presence of ERK2 and mutation of T286 to alanine (T286A) or deletion of the D domain abolished this activity. The siRNA mediated knockdown of CUL1, CUL7 and FBXW8 in HCT116, SW480, U-2 OS and T98 cancer cell lines resulted in the mainly cytoplasmic accumulation of T286 phosphorylated cyclin D1. Under these conditions, the half-life of cyclin D1 was also extended. The nuclear export of phosphorylated cyclin D1 during S phase facilitates its subsequent ubiquitylation by FBXW8 localized within the cytoplasm [9]. Interestingly, cyclin D1 accumulation following CUL1, CUL7 or FBXW8 siRNA was observed to inhibit the proliferation of HCT116 cells and coexpression of inducible T286A mutant abolished this effect. In contrast, knockdown of FBX4 and αB crystallin enhanced the rate of G2 to S phase progression despite the accumulation of cyclin D1. Unlike SCF^FBX4-αB crystallin^, the FBXW8 complex is clearly expressed in various cancer cell lines. Studies on the relative expression levels of FBXW8 in matched tumour samples have not yet been reported [9]. Further studies are thus needed to dissect the relative roles of SCF^FBX4-αB crystallin ^and FBXW8 in various cell lines and tissues.

### Ubiquitin- independent cyclin D1 degradation

The findings of an elegant study on the regulation of cyclin D1 ubiquitylation have recently been reported [27]. Cyclin D1 contains 18 lysines that serve as putative ubiquitylation sites. Feng *et al*., [27] expressed cyclin D1 mutants with single or multiple lysine to arginine substitutions in a human bronchial epithelial cell line. The effects of these mutations on all-*trans*-retinoic acid (RA) (discussed below) induced cyclin D1 polyubiquitylation and degradation were then investigated. In general, single substitutions had little effect on cyclin D1 stability. The joint mutation of lysine 112, 114 or lysine residue 269 alone resulted in a modest increase in cyclin D1 stability but did not prevent polyubiquitylation [27]. The effective abolition of cyclin D1 ubiquitylation required the mutation of at least 17 out of the 18 possible lysine residues. The half-life of these mutants was increased by more than 50 % when *de novo *protein synthesis was inhibited. Of interest is the observation that certain mutations stabilized cyclin D1 but did not affect its polyubiquitylation. Furthermore, a cyclin D1 mutant lacking all lysine residues exhibited a predominantly nuclear localization [27]. These mutants are none the less still susceptible to some form of degradation and their cellular levels are not above those of wild type cyclin D1. In fact, the study by Feng *et al*., (2007) [27] demonstrated that the N- terminal end of cyclin D1 plays an important role in regulating its stability. Ubiquitin independent pathways such as those mediated by antizyme [56] may thus play a crucial role in regulating cellular cyclin D1 levels. The effects of these mutants on cellular proliferation have not been reported.

### Microscopy vs. subcellular fractionation in studies on cyclin D1 proteolysis

Current models on cyclin D1 degradation assume a predominantly nuclear localization with the cytoplasm as the site of degradation. The reader is warned however, that discrepancies exist in regard to the actual cellular localization of cyclin D1. Subcellular fractionation or the selective purification of cellular organelles is a useful method for studying protein localization and redistribution within the cell [71]. Subcellular fractionation techniques have been employed in several studies on cyclin D1 degradation and localization [9,45,72-76]. Intriguingly, these studies suggest that the majority of cyclin D1 is localized within the cytoplasm of various cell lines. Conversely, immunofluorescence studies have consistently demonstrated a predominantly nuclear localization for cyclin D1 outside of S phase [33,40,43,77]. The suggestion that cytoplasmic sequestration regulates cyclin D1 activity in postmitotic neurons, neonatal cardiomyocytes and cancer cell lines is partly based on experiments that involved subcellular fractionation techniques [19,45,75,76]. Importantly, the fractionation of mantle cell lymphoma (MCL) and multiple myeloma (MM) cell lines suggests that cyclin D1b localizes to both the nucleus and cytoplasm [73]. At present, the significance of the observations made in studies using subcellular fractionation remains unclear. It is also unclear if subcellular fractionation techniques can be reliably employed in studies on cyclin D1 localization. It has been proposed that a small protein like cyclin D1 might be released from the nucleus during the preparation of subcellular fractions [54]. The accurate normalization of protein levels or concentrations between different subcellular fractions also presents technical difficulties. A survey of the literature reveals however, that in contrast to cyclin D1 (~34 kDa), the highest levels of small proteins like USF-1 (~34 kDa), c-Fos (~41 kDa), c-Jun (~36 kDa) and CDK2 (~34 kDa) occur in the nuclear fraction [19,71,78]. Cyclin D1 nuclear export also appears to be an active process that requires T286 phosphorylation/CRM1 binding [40] and the protein appears to be immobile within the nucleus [63]. Confocal microscopy analyses also suggested the predominantly cytoplasmic localization of cyclin D1 in MCF-7 cells, even though it appeared to be predominantly nuclear when the same antibody was used for wide-field microscopy [45]. Further studies will be required to validate the use of subcellular fractionation in studies on cyclin D1 localization and degradation. Such validation might be expected to substantially alter our view of how cyclin D1 is regulated at the post translational level in certain cell types.

### Drug induced cyclin D1 degradation

The importance of cyclin D1 in cancer makes it an attractive target for anti-cancer therapy and ablative agents are currently in development [32,79,80]. Several conventional and experimental anti-cancer agents have been observed to induce cyclin D1 degradation in a wide range of cancer cell lines. Additionally, several naturally derived compounds induce cyclin D1 degradation in cancer cells. Cyclin D1 degradation induced by these agents is not strictly dependent on T286 phosphorylation. The literature on the effects of various natural and synthetic compounds on cyclin D1 stability is reviewed below. For clarity, the cell lines, drug concentrations, proteasome inhibitors and rates of cyclin D1 degradation in the various studies are mentioned (see Table 1).

**Table 1:** Compounds known to induce cyclin D1 degradation in mammalian cell lines.

**Compound**	**Conc.**	**T286 dependent? **^**a**^	**Cell line**	**Proteasome inhibitor**
All-*trans *retinoic acid (RA)	4–10 μM	Yes	BEAS-2B, NT2/D1	Lactacystin, LLnL^b^
Differentiation-inducing factor-1 and 3 (DIF-1 and DIF-3)	30 μM	Yes^c^	SCC, HeLa	MG132
1-Methyl-4-phenylpyridinium ion (MPP^+^)	300 nM	Yes	MG63	MG132
hypothemycin	0.5 μg/Ml	N.D.	NIH3T3-DT	Lactacystin
O-methyl deoxybouvardin (RA-VII)	100 nM	N.D.	DLD-1	Lactacystin
GL331	10 μM	N.D.	CL1-5	N-CBZ-L-L-L-AL
Resveratrol	300 μM	N.D.	SW480	LLnL^b^
Diferuloylmethane (curcumin)	25 μM	N.D.	LnCap, various breast cancer derived	Lactacystin
Lovastatin	10 μM		PC-3-M	LLnL^b, d^
Aspirin	5 nmol/L	Yes^e^	SW480, HT-29	MG132
Cycloheximide	50 μM	No	MCF-7	MG132
15-deoxy-Δ^12,14 ^prostaglandin J_2 _(PGJ2)^f^	5–20 μM	N.D.	MCF-7	MG132, PSII
Ciglitazone^f^	30–40 μM	N.D.	MCF-7	MG132, PSII
Troglitazone^f^	40 μM	No	MCF-7	MG132, lactacystin, epoxomicin
Δ2-TG^g^	5 μM	No	MCF-7	MG132, lactacystin, epoxomicin
Rapamycin	100 nmol/L	Yes	MCF-7, MDA-MB-468	LLnL^b^
Trichostatin A (TSA)^h^	1 μM	Yes/no^i^	MCF-7, MDA-MB-231, KNRK	MG132, ALLN, lactacystin, NLVS^j^
Sodium chloride (NaCl), calcium chloride (CaCl_2_), magnesium chloride (MgCl_2_)	50 mM	Yes^e^	Granta 519	LLnL^b^, lactacystin, MG132

a) T286 phosphorylation requirement, b) LLnL (ALLN, Calpain inhibitor 1, N-acetyl-leucyl-leucyl-norleucinal, MG101), c) Mirk/Dyrk 1b mediated T288 phosphorylation required, d) Lactacystin failed to abolish lovastatin induced cyclin D1 degradation, e) p38^SAPK2 ^mediated T286 phosphorylation, f) PGJ2, ciglitazone and troglitazone are PPARγ agonists, g) Δ2-TG is structurally related to troglitazone but lacks PPARγ agonist activity, h) TSA is a prototype HDAC inhibitor, i) partial requirement for GSK3β in TSA induced cyclin D1 degradation, j) NLVS (NIP-leu_3_-vinyl sulphone)

The retinoid receptors comprise a large family of nuclear receptors that regulate various cellular and physiological processes [81,82]. Retinoids inhibit the proliferation of cancer cells and prevent secondary tumour formation. The modulation of retinoid receptor activity thus presents an attractive therapeutic target (reviewed in [31,79]. All-*trans *retinoic acid (RA) has been shown to delay G1/S phase transition and negatively regulate cyclin D1 expression [29]. Treatment of immortalized human bronchial epithelial (BEAS-2B) cells with 4 μM RA results in a rapid decline in cyclin D1 levels within 3–6 h. RA induced cyclin D1 degradation was abolished by specific inhibition of 26S proteasomes using lactacystin. Treatment of NT2/D1 embryonal carcinoma cells with 10 μM RA resulted in reduced cyclin D1 levels within 2 days of treatment [30] and was sensitive to the proteasome inhibitor LLnL (ALLN, Calpain inhibitor 1, N-acetyl-leucyl-leucyl-norleucinal, MG101). RA induced cyclin D1 degradation requires T286 phosphorylation since the T286A mutation abolished polyubiquitylation *in vivo*. It is noteworthy, that RA was considerably more effective at inducing cyclin D1 degradation in BEAS-2B cells (3–6 h) compared to NT2/D1 cells [29,30]. Further studies demonstrated that receptor- nonselective retinoids but not carotenoids mediate the degradation of cyclin D1 via the ubiquitin pathway [83]. The effects of retinoids on cell proliferation and cyclin D1 stability thus appear to be dependent on retinoic acid receptor (RAR) α, RARβ and the retinoid X receptor (RXR) but not RARγ. Retinoid mediated cyclin D1 degradation has been proposed to underlie the chemopreventitive and antiproliferative activity of these compounds [27,29,30].

Differentiation-inducing factor-1 (DIF-1) induces cyclin D1 degradation in human oral squamous cell carcinoma (SCC) and cervical cancer cell lines [84-86]. Differentiation-inducing factors (DIFs) are morphogens that were first identified in *Dictyostelium *where they regulate stalk differentiation [87,88]. DIF-1 induces G0/G1 cell cycle arrest, in part by inducing the ubiquitin-dependent degradation of cyclin D1 [84]. The DIF-1 induced degradation of cyclin D1 is one of the most rapid reported to date, occurring within 30 to 60 minutes after exposure to 30 μM of the compound. Inhibition of proteasome activity with MG132 abolished DIF-1 induced cyclin D1 degradation. DIF-1 activates GSK3β and causes it to relocate to the nucleus. Inhibition or siRNA mediated knockdown of GSK3β attenuated DIF-1 induced cyclin D1 degradation. Similarly, the T286A mutant was resistant to DIF-1 induced degradation, while the T288A mutant was partially resistant. DIF-1 thus induces cyclin D1 degradation in a manner dependent on the phosphorylation of T286 and T288 by GSK3β and Mirk/Dyrk1b respectively. Further studies have demonstrated that DIF-3 (30 μM) induces cyclin D1 degradation in cancer cells similarly to DIF-1. DIF-3 activates GSK3β and Mirk/Dyrk1b in HeLa cells and the induction of cyclin D1 degradation requires phosphorylation of T286 and T288 [85].

MPP^+ ^(1-Methyl-4-phenylpyridinium ion) is an active metabolite of (1-Methyl-4-phenyl-1,2,3,6-tetrahydropyridine (MPTP), that inhibits proliferation and induces cytotoxicity in various cell types [89-91]. MPTP is used to induce experimental models of Parkinson's disease [92,93]. MPP+ induced cell cycle arrest but not apoptosis in MG63 osteosarcoma cells [94]. Doses as low 300 nM induced near complete depletion of cyclin D1 within 4 h of treatment. Co-treatment with the proteasome inhibitor MG132 abolished the MPP^+ ^induced cyclin D1 degradation. MPP^+ ^treatment resulted in the dephosphorylation of Akt and inhibition of GSK3β with LiCl, also effectively abolished cyclin D1 degradation. MPP^+ ^thus induces the ubiquitin-dependent degradation of cyclin D1 in MG63 cells in a phosphorylation dependent manner [94]. The natural metabolites hypothemycin (0.5 μg/Ml) and O-methyl deoxybouvardin (RA-VII) (100 nM) have also been shown to induce the ubiquitin-dependent degradation of cyclin D1 in transformed NIH3T3 mouse and human colon cancer cell lines respectively [95,96]. In both studies, inhibition of proteasomal activity by lactacystin abolished the effect of these compounds on cyclin D1 degradation. GL331 is a semi-synthetic podophyllotoxin derivative similar to etoposide (VP-16) [97,98]. Culture of CL1-5 human lung adenocarcinoma cells with 10 μM GL331 induced cyclin D1 degradation within 4 h of exposure and was sensitive to proteasome inhibition (N-carbobenzyloxy-leucine-leucine-leucine-aldehyde, N-CBZ-L-L-L-AL) [99]. The role of T286 phosphorylation in mediating the degradation of cyclin D1 by hypothemycin, RA-VII and GL331 has not been reported. The phytochemical resveratrol is common in food products including red wine and inhibits cancer cell proliferation *in vitro *[100-102]. Treatment of SW480 colon cancer cells with 300 μM resveratrol induced effective clearance of cyclin D1 within 2 h and was inhibited by LLnL [103]. The effect of resveratrol on cyclin D1 degradation may be cell line specific since the cyclin was not degraded in HL60 promyelocytic leukaemia cells [104]. Diferuloylmethane (curcumin) is a chemopreventitive agent that inhibits the proliferation of a wide range of cancer cells [105,106]. Treatment of LnCap prostate cancer cells and various breast cancer cell lines with 25 μM curcumin for 3 h resulted in a near-total loss of cyclin D1 expression [107]. Co-culture of curcumin with lactacystin abolished cyclin D1 degradation. The role of GSK3β in mediating curcumin induced cyclin D1 degradation however, remains unclear. Curcumin also strongly repressed the expression of cyclin D1 mRNA within 30–60 min in MCF-7 and LnCap cells [107]. The simultaneous repression of cyclin D1 expression and increased degradation at the protein level, partly explains the rapid effect of curcumin on cyclin D1 levels in these cells. Lovastatin inhibits 3-hydroxy-3-methylglutaryl-coenzyme A reductase and has been shown to induce cell cycle arrest [108-110]. Treatment of PC-3-M prostate cancer cells with 10 μM Lovastatin for 36 h induced cyclin D1 degradation. Lovastatin induced cyclin D1 degradation was inhibited by LLnL but not lactacystin [111]. Aspirin [112,113] was recently reported to induce the p38^SAPK2 ^dependent degradation of cyclin D1 in SW480 and HT-29 colon cancer cells [114]. Treatment of SW480 cells with 5 nmol/L aspirin induced complete clearance in about 1 h. The effect of aspirin on cyclin D1 stability in HT-29 cells was less potent, inducing a partial reduction of protein levels within 4 h. Aspirin induced cyclin D1 degradation was effectively inhibited by MG132. As expected, inhibition of p38^SAPK2 ^activity abolished aspirin induced cyclin D1 degradation [114].

Cycloheximide inhibits protein synthesis by preventing peptide initiation and extension in ribosomes [115,116]. Although it is not a therapeutic agent, cycloheximide is commonly used in studies on protein stability and degradation. In most cell lines, cyclin D1 levels rapidly decline following the inhibition of protein synthesis with cycloheximide. MG132 effectively abolishes cyclin D1 degradation following the inhibition of protein synthesis [8,26,27]. Treatment of MCF-7 cells expressing cyclin D1 constructs lacking lysine residues with cycloheximide still resulted in a decline in protein levels [27]. This observation highlights the importance of ubiquitin independent degradation in regulating cyclin D1 stability.

The proliferator- activated receptor (PPAR) superfamily of nuclear receptors mediates cellular processes such as proliferation and differentiation (reviewed in [117-119]). Activation of the proliferator- activated receptor γ (PPARγ) by 15-deoxy-Δ^12,14 ^prostaglandin J_2 _(PGJ2) or synthetic ligands, inhibits tumour cell proliferation and induces the ubiquitin dependent degradation of cyclin D1 [120,121]. Treatment of MCF-7 cells with 5–20 μM PGJ2 or 30–40 μM or the thiazolidinedione based PPARγ agonist ciglitazone inhibited G1 to S phase progression but not apoptosis. Following treatment with 30 μM PGJ2 or 80 μM ciglitazone, total cyclin D1 levels were reduced to undetectable levels between 3 and 15 h after addition of either compound [120]. Cyclin D1 degradation was inhibited by the proteasome inhibitor MG132 (carbobenzyloxy-leucyl-leucyl-leucinal, Z-LLL-CHO) and PSII but not calpain inhibitors and resulted in the accumulation of poly-ubiquitylated species. Indirect fluorescence microscopy analyses suggested that cyclin D1 accumulates in the cytoplasm of PGJ2 treated MCF-7 cells [120]. This study did not however, investigate the role of GSK3β in mediating PGJ2 or ciglitazone induced cyclin D1 degradation. Later studies by Huang *et al*., [28], have demonstrated that the effect of synthetic PPARγ agonists on cyclin D1 stability is independent of PPARγ activation. In this study, a related PPARγ agonist, troglitazone (at concentrations of 40 μM), induced cyclin D1 degradation in MCF-7 cells 12–18 h after exposure. Co treatment of MCF-7 cells with the PPARγ antagonist GW9662 did not suppress troglitazone induced cyclin D1 degradation. Furthermore, additional thiazolidinediones such as rosiglitazone and piolitazone did not affect cyclin D1 stability, while derivatives that have no effect on PPARγ activity still induced degradation. Similarly to ciglitazone [120], troglitazone induced cyclin D1 degradation was also sensitive to specific inhibition of 26S proteasomes by MG132, lactacystin and epoxomicin. Troglitazone did not however, activate GSK3β or require the activity of this kinase to induce cyclin D1 degradation. Derivatives of ciglitazone and troglitazone (Δ2-TG and Δ2-CG) that are inactive as PPARγ activators also induced cyclin D1 degradation. The effect of these derivatives on cyclin D1 stability was similar to that of their parent compounds with slightly enhanced potency. The modification and optimization of Δ2-TG structure (by substitution of the terminal hydroxyl moiety) has resulted in the synthesis of derivatives with improved potency, capable of inducing cyclin D1 degradation at concentrations as low as 5 μM [80]. Taken together, the studies by Wang *et al*., Qin *et al*. and Huang *et al*., [80,120,121] indicate that PGJ2 and some synthetic PPARγ agonists induce the ubiquitin-dependent degradation of cyclin D1 independently of GSK3β activity. Since Δ2-TG derivatives induce cyclin D1 degradation at low concentrations that are clinically achievable independently of PPARγ activation, Huang *et al*., [80] have suggested that these compounds may be particularly useful as a platform for developing therapeutic cyclin D1 ablative agents.

Mammalian target of rapamycin (mTOR) is a target of the phosphatidylinositol 3-Kinase (PI3K)/protein kinase B (PKB/Akt) pathway (reviewed in [121-126]) and plays a major role in coupling mitogenic stimuli to cell cycle progression. Rapamycin binds to FK506 binding protein 12 (FKBP12) to form a complex which binds to and inactivates mTOR [127-130]. mTOR regulates several cellular processes. Previous studies have shown that mTOR regulates protein synthesis by phosphorylating the 4E binding protein1 (4E-BP1), which frees the eukaryotic initiation factor-4 (eIF-4F) to form a multi-subunit complex with eIF-4A, eIF-4B and eIF-4G. The formation of active eIF4 complexes results in an increase in the rate of translation of cyclin D1 mRNA and other regulators of G1- S phase progression [123]. Deregulated PKB/Akt activity is common in many cancers and results in mTOR mediated stimulation of G1- S phase progression. The activity of several anti-apoptotic proteins that promote cancer cell survival is also increased as a result of deregulated PKB/Akt activity [131-133]. Furthermore, the deregulation of mTOR activity is associated with the development of both solid and haematological malignancies (reviewed in [134]). PKB/Akt and mTOR thus present important therapeutic targets for anti-cancer agents [135]. Rapamycin inhibits cellular proliferation and induces G1 arrest, partly by repressing the expression of cyclin D1 [136-139]. In addition to suppressing the translation of cyclin D1 mRNA [137], rapamycin also induces the ubiquitin-dependent degradation of cyclin D1 [136]. Treatment of MCF-7 cells with 100 nmol/L rapamycin resulted in an approximately 50 % reduction in cyclin D1 levels. In MDA-MB-468 breast cancer cells which express lower levels of cyclin D1, treatment with this concentration of rapamycin reduced the level of cyclin D1 to near undetectable levels within 6 h. Treatment with rapamycin also reduced the half life of cyclin D1 in MCF-7 and MDA-MB-468 cells by approximately 44 and 20 % respectively. Inhibition of 26S proteasomal activity with ALLN abolished rapamycin induced cyclin D1 degradation. Rapamycin also activated GSK3β although this was not accompanied by phosphorylation of serine 9 (Ser9). Inhibition of GSK3β in MCF-7 cells by LiCl, SB216763 and SB415286 abolished cyclin D1 degradation as effectively as ALLN. Similarly, an exogenously expressed cyclin D1 T286A mutant was refractory to rapamycin induced degradation [136]. Taken together, these findings demonstrate that rapamycin activates GSK3β and induces the phosphorylation dependent degradation of cyclin D1 via the ubiquitin pathway in breast cancer cell lines.

Histone deacetylase inhibitors (HDACIs) have shown potent selective activity as anti cancer agents both *in vitro *and *in vivo *[140-146]. Trichostatin A (TSA) is a prototype HDACI that has been shown to induce cyclin D1 degradation in human breast cancer and transformed rat kidney fibroblast cell lines. Treatment of MCF-7 cells with 1 μM TSA induced complete cyclin D1 degradation within 6 h of drug addition [25]. Similar results were obtained in MDA-MB-231 and rat KNRK cells, although the effect of TSA on cyclin D1 stability was less pronounced in these cell lines [25,45]. A structurally unrelated HDACI, HC-toxin (HCT) also repressed cyclin D1 expression in MCF-7 and MDA-MB-231 cells [25]. The induction of cyclin D1 degradation may thus be a general feature of HDACIs. TSA induced the accumulation of polyubiquitylated cyclin D1 species and the nuclear export of the recombinant wild type protein but not a T268A mutant. The proteasome inhibitors MG132, ALLN, lactacystin and NLVS (NIP-leu_3_-vinyl sulphone) all inhibited TSA induced cyclin D1 degradation to varying degrees in MCF-7 cells. The inhibition of calpain activity with ALLM (Calpain inhibitor II, N-acetyl-leucyl-leucyl-methional) or cathepsin activity did not affect TSA induced cyclin D1 degradation. TSA also induced accumulation of SKP2 in MCF-7 cells. Furthermore, the siRNA mediated knockdown of SKP2 delayed TSA induced cyclin D1 degradation [25]. The recent identification of *bona fide *E3 ligase complexes that target cyclin D1 suggests however, that SKP2 is not directly involved in the degradation of this cyclin [8,9]. It remains to be determined if SKP2 indirectly affects cyclin D1 stability in TSA treated MCF-7 cells. Inhibition of GSK3β activity with SB216763 or knockdown by siRNA partially inhibited cyclin D1 degradation. Similarly, inhibition of CRM1 mediated nuclear export with leptomycin B (LMB) also partially abolished TSA induced degradation. The ubiquitin-dependent degradation of cyclin D1 induced by TSA in MCF-7 cells is thus partially dependent on GSK3β activity [26]. HDACIs have also been shown to delay entry into mitosis by activating the p38^SAPK2 ^checkpoint [50]. Although osmotic stress has been shown to induce the p38^SAPK2^-dependent degradation of cyclin D1, the role of p38^SAPK2 ^in mediating HDAC induced cyclin D1 degradation remains to be investigated. HDACIs have recently been shown to sensitize cancer cells to the cytotoxic effects of conventional therapeutic agents [147-152]. The effect of HDACIs on cyclin D1 stability may underlie some of these effects. Deregulated cyclin D1 expression has been associated with resistance to endocrine and erbB therapies [20-22,153,154]. HDACIs may thus be clinically useful in overcoming drug resistance associated with cyclin D1 over expression.

Pharmacologically and structurally diverse compounds have been shown to induce cyclin D1 degradation. Little is known about how these compounds affect the stability of the cyclin D1b variant which lacks the c-terminal regulatory sequences. PPARγ activators and HDAC inhibitors have been shown to induce cyclin D1 degradation independently of T286 phosphorylation. These compounds may thus also be useful in the ablation of cyclin D1b levels.

### Unresolved questions

The recent identification of SCF-complexes that mediate cyclin D1 degradation has greatly broadened the understanding of how the stability of this cyclin is regulated [8,9]. Prior studies suggest however, that additional mediators of cyclin D1 degradation exist [56]. Both SCF^FBX4-αB crystallin ^and FBXW8 specifically target T286 phosphorylated cyclin D1 for ubiquitin-dependent degradation. Cyclin D1 degradation can occur independently of T286 phosphorylation and variants that lack threonine phosphorylation sites are still relatively unstable [46,63]. In MCF-7 cells, the expression of αB crystallin is extremely low or absent [8]. None the less, inhibition of protein synthesis with cycloheximide still results in the fairly rapid decline of cyclin D1 levels [8]. Inhibition of GSK3β or CRM1 dependent nuclear export failed to abolish the rapid degradation of cyclin D1 [26]. Cyclin D1 can thus be effectively degraded even in the absence of SCF^FBX4-αB crystallin ^complexes. Several potential anti-cancer agents have been shown to induce cyclin D1 degradation via the proteasome (discussed above). The effects of these agents on the expression and activity of SCF^FBX4-αB crystallin ^and FBXW8 remains to be determined. SCF^FBX4-αB crystallin ^expression is often lost in cancer cells as a result of chromosomal deletions [8]. It is thus unclear if drug induced cyclin D1 degradation is dependent on the expression of SCF^FBX4-αB crystallin^, FBXW8 and other specific E3 ligases. PPARγ agonists and HDAC inhibitors effectively induce cyclin D1 degradation even in the absence of GSK3β activity [26,28]. Since SCF^FBX4-αB crystallin ^and FBXW8 specifically target T286 phosphorylated cyclin D1, it is possible that the degradation induced by troglitazone and TSA occurs independently of these E3 ligases. The precise roles of both complexes in mediating cancer cell proliferation remain unclear. Knockdown of SCF^FBX4-αB crystallin ^complexes by siRNA shortened progression into S phase following nocodazole release. Furthermore, SCF^FBX4-αB crystallin ^expression is frequently lost in cancer cell lines and primary cancers [8]. In direct contrast, siRNA mediated knockdown of FBXW8 complexes inhibited the proliferation of cancer cell lines [9]. SCF^FBX4-αB crystallin ^and FBXW8 may thus regulate different aspects of phosphorylation dependent cyclin D1 degradation. It is also possible that while the loss of SCF^FBX4-αB crystallin ^allows the accumulation of cyclin D1 during cancer development, the continued expression of FBXW8 is required to maintain levels below the threshold required to repress DNA replication [38,43,54].

An early study suggested a role for calpains in regulating cyclin D1 stability [111]. In this study, LLnL and ALLM but not lactcystin inhibited serum starvation induced cyclin D1 loss in NIH 3T3 cells. Furthermore, lactacystin failed to abolish actinomycin D and lovastatin induced cyclin D1 loss in PC-3-M prostate cancer cells. For these reasons, cyclin D1 stability was initially assumed to be regulated by calpains (ref). MG132 completely abolished TSA induced cyclin D1 degradation in MCF-7 cells [26,45]. Lactacystin only partially inhibited TSA induced cyclin D1 degradation but completely abolished troglitazone induced cyclin D1 degradation [28]. The inhibition of protein synthesis with cycloheximide led to the rapid decline of cyclin D1 levels in MCF-7 cells. This loss of cyclin D1 expression was inhibited by MG132 but not lactacystin, SB216763 or LMB [26]. Ubiquitin-dependent degradation clearly plays an important role in regulating cyclin D1 stability [8,9]. It is possible however, that peptide aldehyde proteasome inhibitors like MG132 and LLnL block additional pathways that regulate cyclin D1 stability [155-157].

The SK-UT-1B cell line presents an interesting paradox. The ubiquitin-dependent degradation of cyclin D1 is defective in this cell line and lysates fail to mediate the polyubiquitylation of cyclin D1 *in vitro *[46,158]. In contrast to MCF-7 cells, the inhibition of protein synthesis did not lead to the rapid decline of cyclin D1 levels in SK-UT-1B cells. Furthermore, cyclin D1 levels remained elevated even when the cells were arrested at the G1/S phase boundary with hydroxyurea [45]. These observations suggest that cyclin D1 levels do not decline during S phase in this cell line. The expression of a SKP2 splice variant which fails to migrate to the nucleus has been linked to defective proteolysis in SK-UT-1B cells [158], since expression of SKP2 rescued cyclin D1 degradation. Recent studies suggest however, that SKP2 is not a direct mediator of cyclin D1 degradation [8,9]. SK-UT-1B cells may thus harbour additional defects in pathways that regulate cyclin D1 degradation. The degradation of cyclin D1 at S phase facilitates DNA replication and cell cycle progression. In fact, deregulated cyclin D1 expression is known to inhibit cell proliferation and the generation of clones stably expressing the T286A mutant is somewhat difficult [9,38,43,54]. The question thus arises: how SK-UT-1B cells proliferate in the absence of cyclin D1 degradation? One possibility is that SK-UT-1B cells integrate the proliferative and anti-proliferative properties of cyclin D1 by means of cytoplasmic sequestration. In this model, cyclin D1 is retained in the cytoplasm and its nuclear import is dependent on its binding partners (CDK4, CDK6, p21, p27 and various transcription factors). At the same time, nuclear accumulation is tightly regulated by the continuous nuclear export of GSK3β phosphorylated cyclin D1 throughout the cell cycle [44,45]. The nuclear accumulation of cyclin D1 is thus limited by its continuous export which prevents PCNA and CDK2 inhibition. This would permit the effective replication of DNA in the absence of cyclin D1 degradation. The simultaneous inhibition of ubiquitin-dependent degradation and nuclear export did not result in the nuclear localization of cyclin D1 when its synthesis was inhibited in MCF-7 and SK-UT-1B cells [45]. The precise mechanism(s) that underlie the defective degradation of cyclin D1 in SK-UT-1B cells remains to be clearly defined. Such characterization could provide further insights into the regulation of cyclin D1 stability.

GSK3β activity appears to remain unchanged throughout the cell cycle and recent studies suggest a limited role for this kinase in mediating cyclin D1 degradation [44]. Inhibition of GSK3β can however, inhibit TSA induced cyclin D1 degradation and results in increased levels of the cyclin within the nucleus [26,45]. Changes to the accessibility of cyclin D1 during S phase could also result in increased T286 phosphorylation in the absence of increased GSK3β activity [43,44]. If its major role does not involve regulating cyclin D1 stability, what then is the cellular function of GSK3β mediated T286 phosphorylation? The transforming activity of the T286A mutant and cyclin D1b seems to depend more on their constitutive nuclear localization, than on their stability or capacity to phosphorylate RB [63]. It is possible therefore, that GSK3β functions to regulate the levels of cyclin D1 within the nucleus. Cyclin D1 has been reported to be sequestered in the cytoplasm of post mitotic neurons [75]. The cytoplasmic sequestration of cyclin D1 may also present also a novel mechanism for regulating its activity in some cancer cell lines [45]. It remains to be seen, whether the concept of a predominantly cytoplasmic localization for cyclin D1 turns out to be a paradigm or a heresy.

## Conclusion

Cyclin D1 is an important regulator of cell cycle progression and overexpression of cyclin D1 has been linked to the development and progression of cancer. Deregulated cyclin D1 expression is also linked to the development of resistance to hormone therapy in breast cancer. In many cancers, the impaired ubiquitin-dependent degradation of cyclin D1 is responsible for its elevated levels. A number of therapeutic agents have been shown to induce cyclin D1 degradation via the ubiquitin pathway. Drug induced cyclin D1 ablation may provide a useful chemopreventive or treatment strategy for cancer. The development of such agents requires a firm understanding of cyclin D1 regulation. Recent reports have increased our understanding of how cyclin D1 degradation is regulated. At present, a number of questions remain unanswered. These include the role of phosphorylation and/or ubiquitin-independent degradation, the effects of agents that induce cyclin D1 degradation on the stability and/or activity of cyclin D1b, SCF^FBX4-αB crystallin ^and FBXW8, as well as the precise role of GSK3β in regulating cyclin D1 activity. Finally, the significance of cyclin D1 sequestration within the cytoplasm needs to be properly addressed. We can look forward to further exciting developments in this important area of cancer cell research in the coming years.

## Competing interests

The author(s) declare that they have no competing interests.

## Authors' contributions

JPA reviewed the literature, drafted and finalized the manuscript.
